# LesR is a novel upstream regulator that controls downstream Clp expression to modulate antibiotic HSAF biosynthesis and cell aggregation in *Lysobacter enzymogenes* OH11

**DOI:** 10.1186/s12934-017-0818-2

**Published:** 2017-11-14

**Authors:** Huiyong Xu, Ruping Wang, Yangyang Zhao, Zheng Qing Fu, Guoliang Qian, Fengquan Liu

**Affiliations:** 1 Institute of Plant Protection, Jiangsu Academy of Agricultural Sciences, Nanjing, 210014 People’s Republic of China; 2 College of Plant Protection, Nanjing Agricultural University, China/Key Laboratory of Integrated Management of Crop Diseases and Pests (Nanjing Agricultural University, Ministry of Education), Nanjing, 210095 People’s Republic of China; 3 Department of Biological Sciences, University of South Carolina, Columbia, SC 29208 USA

**Keywords:** *Lysobacter enzymogenes*, Clp, LesR, HSAF, Cell aggregation

## Abstract

**Background:**

Heat-stable antifungal factor (HSAF) is a polycyclic tetramate macrolactam secondary metabolite that exhibits broad-spectrum inhibitory activities against filamentous fungal pathogens. The native yield of this chemical is low. It is also a great challenge to synthesize HSAF artificially, due to its complex structure. Understanding the regulatory mechanism underlying HSAF biosynthesis could provide genetic basis for engineering high HSAF-producing strain. The transcription factor Clp is a global regulator that controls bacterial pathogenicity and the expression of one hundred related genes in the phytopathogenic bacterium *Xanthomonas campestris* pv. *campestris* (*Xcc*). Diffusible signal factor (DSF) chemical signaling is the only well-characterized upstream regulatory pathway that involves downstream Clp regulation in *Xcc*. Such a regulatory hierarchy between DSF signaling and Clp is also conserved in the Gram-negative biological control agent *Lysobacter enzymogenes*, where the DSF signaling system controls antifungal antibiotic HSAF biosynthesis via Clp.

**Results:**

Here, using *LLysobacter enzymogenes* OH11 as a working organism, we examined a novel upstream regulator, LesR, a LuxR solo that controls Clp expression to modulate HSAF biosynthesis as well as cell aggregation. We found that the overexpression of *lesR* in strain OH11 almost entirely shut down HSAF production and accelerated cell aggregation. These changed phenotypes could be rescued by the introduction of plasmid-borne *clp* in the *lesR* overexpression background. Consistent with findings, we further found that overexpression of *lesR* led to a decrease in the Clp level.

**Conclusions:**

These results collectively have shown that LesR could exert its function, i.e., HSAF biosynthesis, via downstream Clp. These findings were subsequently validated by a comparative transcriptome analysis, where the regulatory action of LesR was found to largely overlap with that of Clp. Therefore, in addition to the well-known DSF signaling system, the present study reveals that LesR functions as a new upstream regulatory factor of Clp in *L. enzymogenes*. The key factor was important for the production of HSAF. The strains with high HSAF yield can presumably be constructed by deletion of the negative regulators or overexpression of the positive regulators by genetic engineering.

**Electronic supplementary material:**

The online version of this article (10.1186/s12934-017-0818-2) contains supplementary material, which is available to authorized users.

## Background

*Lysobacter* is a Gram-negative genus in the family of Xanthomonadaceae and is one of the most ubiquitous environmental microorganisms [1]. A representative and well-studied species of this genus is *Lysobacter enzymogenes*, which is recognized as one of the important biological control agents against diverse fungal pathogens through the production of abundant extracellular lytic enzymes and the secondary antifungal antibiotic heat-stable antifungal factor (HSAF) [1–5]. Because of HSAF’s complex structure, it is very difficult to synthesize HSAF artificially. Previous studies have shown that the production of HSAF by *L. enzymogenes* was low. Due to the large gene cluster of PKS/NRPS (HSAF Synthetics gene cluster), it is difficult to express these genes in a heterogenous system such as *Escherichia coli*. Therefore it is imperative to elucidate the regulatory network that controls HSAF biosynthesis. The key factor LesR has been shown to be important for the production of HSAF. The strains of high yield HSAF were constructed by deletion of negative regulators or overexpression of positive regulators by genetic engineering. The production of HSAF is effectively increased and it is a key part of industrialization.

Clp, a cAMP-receptor-like protein, is recognized as a global regulator possessing an N-terminal cNMP (cyclic nucleotide monophosphate) domain and a C-terminal HTH (helix-turn-helix)-DNA binding domain in the phytopathogenic bacterium *Xanthomonas campestris* pv. *campestris* (*Xcc*) [6, 7]. In this bacterium, Clp both directly and indirectly controls a wide range of important physiological and cellular processes, including EPS (extracellular polysaccharide) biosynthesis, the production of plant-cell-degrading enzymes, cell motility, biofilm formation and virulence [6]. In the direct mode, *Xcc* Clp modulates the transcription of target genes by directly binding to their promoters, which is released when *Xcc* Clp is bound by c-di-GMP, a newly identified nucleotide second messenger in bacteria [7, 8]. *Xcc* Clp is found to be a receptor for c-di-GMP binding via its N-terminal cNMP domain [7]. It was also found that regulation via Clp is dynamically influenced by the small-molecule chemical diffusible signal factor (DSF) in *Xcc*, whose biosynthesis and signal transduction are dependent on a *rpf* gene cluster [9–11]. In essence, *rpfF* encodes a putative enoyl-CoA hydratase involved in synthesizing DSF, whereas the RpfC (histidine kinase)/RpfG (response regulator) two-component system is responsible for DSF sensing and signal transduction, respectively [9, 11]. When the DSF signal is first sensed by membrane-bound RpfC, it becomes free and activates the coupled RpfG, which is an HD-GYP phosphodiesterase that degrades c-di-GMP to GMP [12, 13]. In the absence of c-di-GMP, Clp is free to bind to a variety of its cognate promoters, enabling the regulation of a significant number of downstream genes [6, 7, 12]. These previous data suggest that Clp plays an important role in the *Xcc* DSF signaling pathway. Indeed, the *Xcc* DSF signaling system was found to be significantly involved in regulating *Xcc* pathogenicity by controlling the production of a variety of virulence factors via Clp [6, 10]. Furthermore, it is also important to note that *Xcc* DSF signaling appears to be the only well-characterized upstream regulatory pathway that involves downstream Clp regulation in bacteria, although both of them are widely conserved in *Xanthomonas* and its close genera, such as *Xylella* and *Lysobacter* [6, 8, 14, 15].

The Clp homologue in *L. enzymogenes* controls HSAF biosynthesis and the production of diverse lytic enzymes, cell adherence/aggregation and antifungal activity [2, 15]. Recently, a new DSF family signaling molecule, *Le*DSF3 (13-methyltetradecanoic acid), was shown to modulate HSAF biosynthesis via the conserved RpfC/RpfG two-component system and the global regulator Clp in *L. enzymogenes* [2, 16]. These results strongly suggest that the regulator Clp serves as a downstream component of DSF signaling and controls HSAF biosynthesis in *L. enzymogenes*, which was recently confirmed by our laboratory and collaborators [16, 17]. However, Clp also controls other vital physiological functions, such as cell aggregation in a DSF-independent manner in *L. enzymogenes*, since the disruption of DSF signaling did not change cell adherence/aggregation, as shown in the *clp* mutant [2, 14]. These findings point out the possibility that a DSF-independent and a previously unidentified upstream regulator might control cell adherence/aggregation through Clp in *L. enzymogenes*.

Interestingly, a LuxR solo, LesR, was recently shown to control cell aggregation and HSAF production in *L. enzymogenes* OH11 [18]. LesR contains an N-terminal AHL (*N*-acyl homoserine lactone) domain and a C-terminal HTH DNA binding domain [18]. The domain organization of LesR is highly similar to that of the LuxR proteins in the typical LuxI/LuxR QS (Quorum sensing) system, but *L. enzymogenes* OH11 does not seem to contain the corresponding LuxI protein [18]. These LuxR proteins were thus termed LuxR orphans or solos [19, 20]. Importantly, overexpression, but not deletion, of *lesR* was found to accelerate cell aggregation and almost entirely impair HSAF production in wild-type OH11 [18]. These LesR-controlled phenotypes are similar to those controlled by Clp, establishing a bridge to functionally connect Clp and LesR in *L. enzymogenes*, although their genetic relationship is still unclear to date.

In the present study, we found that LesR is a novel upstream regulator of Clp in *L. enzymogenes*. Our genetic, biochemical and transcriptomic analyses show that one of the mechanisms by which LesR regulates cell aggregation and HSAF biosynthesis is to control Clp expression in *L. enzymogenes*. Therefore, in addition to the well-characterized DSF signaling, our studies from *L. enzymogenes* provide information on a new upstream regulatory factor, LesR, whose functional regulation is mediated by the downstream Clp levels in bacteria.

## Results

### LesR is involved in the regulation of cell aggregation and HSAF biosynthesis via Clp

**Fig. 1:**
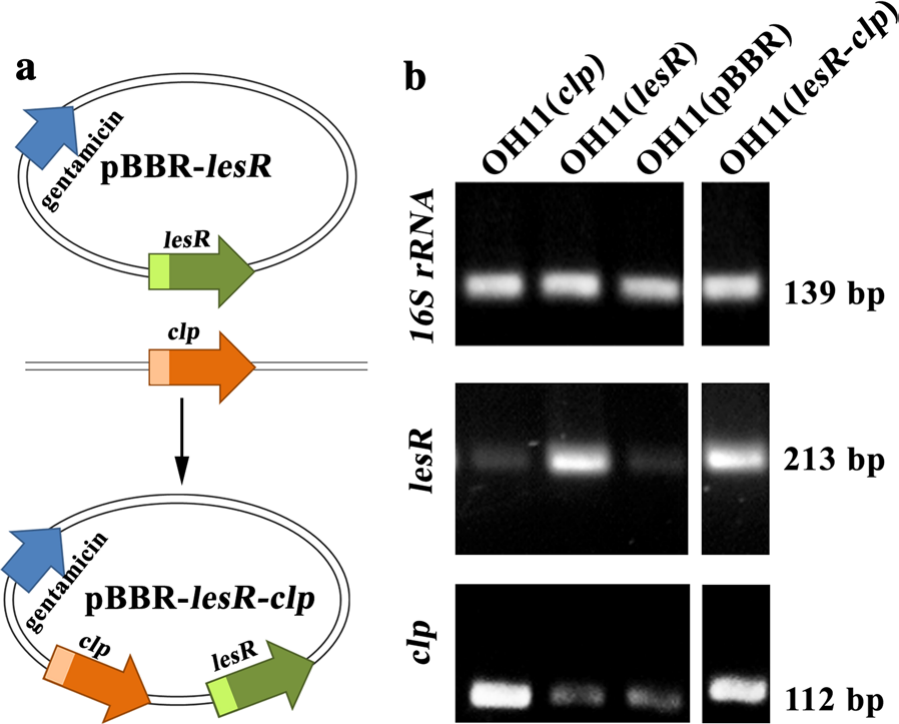
Introduction of *clp* into the overexpression strain of *lesR*. **a** Plasmid construction leading to overexpression of *clp* under the control of its native promoter in the background of *lesR* overexpression. **b** RT-PCR assay of *lesR* or *clp* in the respective transformed *L. enzymogenes* strain. The gene *16S rRNA* was used as an internal control as described previously [14]. OH11(pBBR), the wild-type strain with an empty vector; OH11(*lesR*), the *lesR* overexpression strain; OH11(*clp*), the *clp* overexpression strain; and OH11(*lesR*-*clp*), the introduction of *clp* into OH11(*lesR*)

**Fig. 2:**
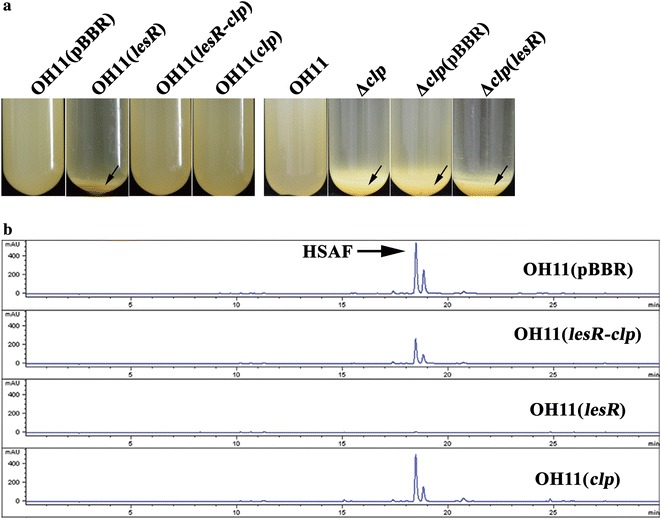
Introduction of *clp* into the strain overexpressing *lesR* eliminated cell aggregation (**a**) and restored the HSAF levels (**b**). The arrows indicate the aggregated cells located at the bottom of the tube. The characterized HSAF is indicted by an arrow in part **b**. OH11, the wild-type strain; Δ*clp*, the *clp* deletion mutant; Δ*clp*(pBBR), Δ*clp* containing an empty vector; and Δ*clp*(*lesR*), Δ*clp* with overexpressed *lesR*. OH11(*lesR*), the *lesR* overexpression strain; OH11(*clp*), the *clp* overexpression strain; and OH11(*lesR*-*clp*), the introduction of *clp* into OH11(*lesR*)

To explore the genetic relationship between LesR and Clp, we first overexpressed *clp* in the *lesR* overexpression background and did a cell aggregation assay of the transformed strains. As shown in Fig. 1a, the coding region of *clp* under the control of its native promoter was successfully inserted into the vector pBBR-*lesR*, and it was previously shown to possess the intact *lesR* gene under the control of its native promoter [18]. As shown in Fig. 1b, the overexpressed transcript of *clp* or *lesR* was validated by RT-PCR in the corresponding transformed strains. Next, these transformed strains were subjected to a cell aggregation assay. It was clear that OH11(*lesR*), the *lesR* overexpression strain, displayed the expected cell aggregation behavior in LB broth, in agreement with our earlier report [18]. However, under similar assay conditions, the introduction of *clp* eliminated the cell aggregation of OH11(*lesR*) (Fig. 2a). Furthermore, the overexpression of *clp* in the wild-type OH11 did not cause cell aggregation, which was similar to that of wild-type OH11 containing an empty vector (Fig. 2a). These results suggest that Clp was genetically situated downstream of the LesR regulatory pathway to control cell aggregation. Consistent with these data, the overexpression of *lesR* could not rescue the deficiency of the *clp* mutant in terms of cell aggregation (Fig. 2a).

Next, we investigated whether Clp also serves as a downstream regulator to modulate LesR-controlled HSAF biosynthesis. To address this point, the HSAF levels from various *Lysobacter* strains described above were determined by HPLC. As shown in Fig. 2b, the *lesR* overexpression strain OH11(*lesR*) did not produce any detectable HSAF, in accordance with our previous report [18]. Surprisingly, it was obvious that the overexpression of *clp* partially restored the deficiency of OH11 (*lesR*) to produce the wild-type HSAF yield (Fig. 2b). However, overexpression of *clp* in the wild-type OH11 did not change the yield of HSAF, which was similar to that of the wild-type strain containing an empty vector (Fig. 2b). These results also suggest that involvement of the LesR regulatory pathway in the regulation of HSAF biosynthesis occurs, at least in part, through the downstream factor Clp in *L. enzymogenes*.

### The protein accumulation of Clp is regulated by LesR

**Fig. 3:**
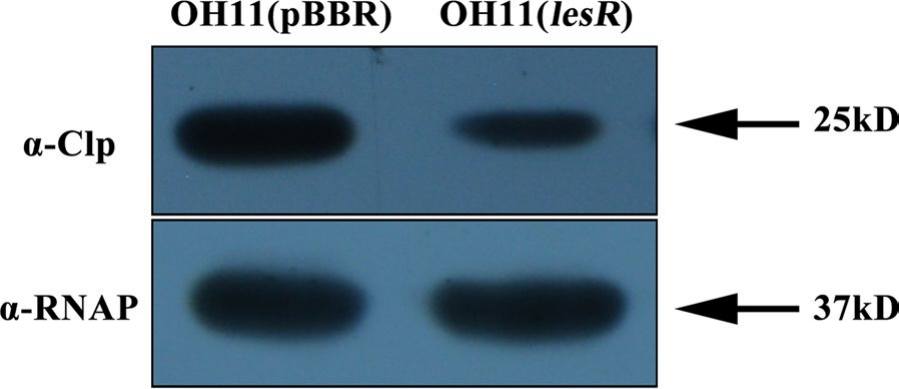
The protein level of Clp was regulated by LesR. A 25-kDa band corresponding to the predicated size of Clp was detected by the polyclonal antibody α-Clp in the control strain OH11(pBBR). The amount of this band appears to have decreased levels in the *lesR* overexpression strain OH11(*lesR*). The RNA polymerase α-subunit (37 kDa) was used as the internal control for loading, which was detected by the specific antibody α-RNAP

Since overexpression of *clp* restored the phenotypic changes in the OH11(*lesR*) strain in terms of cell aggregation and HSAF yield, we thus speculated that overexpression of *lesR* probably decreased the protein level of Clp in *L. enzymogenes*. To test this, a western blot assay was performed for the wild-type OH11 containing the overexpressed *lesR*, named OH11(*lesR*) and an empty vector, named OH11(pBBR). As shown in Fig. 3, we found that a band (25 kDa) corresponding to the predicted size of Clp was clearly detected in the control strain, which was the wild-type OH11 possessing an empty vector. In comparison, Clp protein level was significantly reduced in the *lesR* overexpression strain under similar test conditions. Furthermore, the internal control (the α subunit of the RNA polymerase) was detected at similar levels, both in the control strain and the *lesR* overexpression strain. These results collectively indicate that overexpression of *lesR* impaired the protein accumulation of Clp in *L. enzymogenes*.

### The regulatory action of LesR largely overlaps with that of Clp

**Fig. 4:**
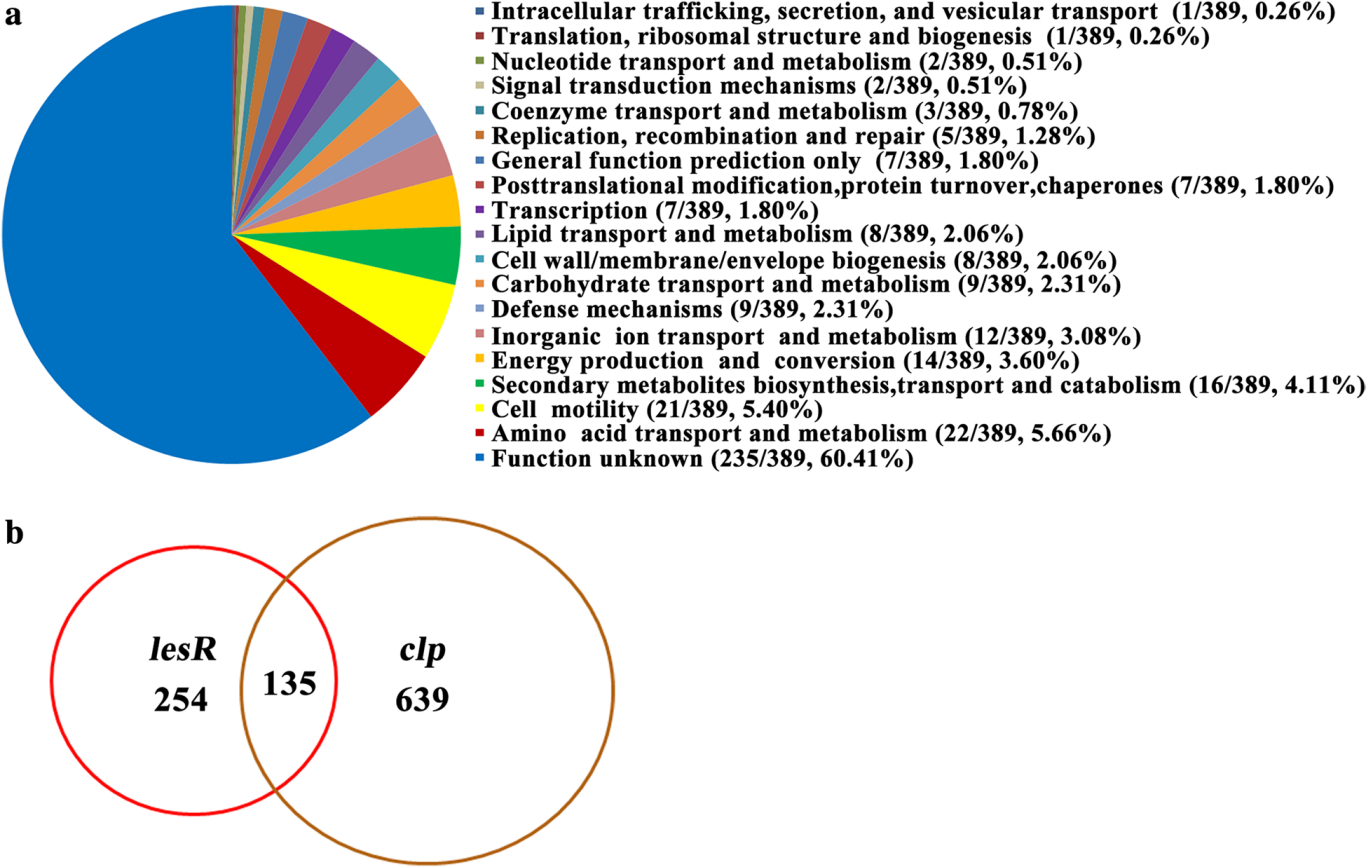
LesR and Clp regulated a significant number of overlapping transcripts. (**a**) RNA-Seq revealed that LesR has a broad regulatory effect, controlling the transcription of 389 genes (Additional file 1: Table S2). The predicted functions of the LesR-controlled gene products are indicated by different colors. **b** The overlap of LesR and Clp in their controlled transcripts. A total of 135 genes (Additional file 1: Table S2) were found to be controlled at the mRNA level both by LesR and Clp. The data of the transcriptome of Clp were obtained from our published report [15]

To further validate the functional link between LesR and Clp, the effect of *lesR* at the transcriptomic level was determined by RNA-Seq. The results showed that overexpression of *lesR* had a broad effect on the transcriptome, significantly influencing the expression of 389 genes (Fig. 4a; Additional file 1: Table S2). These LesR-regulated genes were associated with 19 functional groups, including secondary metabolites and cell envelope biogenesis (Fig. 4a). In agreement with the decreased amount of HSAF, the expression of four key genes within the HSAF biosynthetic cluster was significantly decreased in the *lesR* overexpression strain in comparison to the control strain (Additional file 1: Table S2). Comparative transcriptome analysis revealed that 135 genes from the pool, whose expression was controlled by *lesR*, were also influenced by Clp, including the characterized HSAF biosynthetic genes. A number of gene homologues from the LesR and Clp regions, i.e., *pilX1* and *pilV1*, have been shown to control cell aggregation in *L. enzymogenes* (data not shown), providing an additional piece of evidence to support our findings in the present study that LesR and Clp both control cell aggregation. These results indicate that a considerable amount of overlap in the gene expression profiles between these two factors (LesR and Clp) exists. Collectively, the above phenotypic and gene expression data support the notion that Clp serves as a downstream factor of the LesR regulatory pathway to modulate a number of LesR-controlled functions, such as HSAF biosynthesis and cell aggregation. However, it was also clearly observed that Clp regulated the expression of a unique set of 639 genes in a LesR-independent manner in *L. enzymogenes* (Fig. 4b; Additional file 1: Table S2).

## Discussion

In the present study, we provide the first report that Clp serves as a downstream factor of the LesR regulatory pathway to control HSAF biosynthesis and cell aggregation in *L. enzymogenes*, in addition to the characterized DSF signaling pathway [6, 17]. This knowledge adds new insights into the complex regulatory pathways that involve the global regulator Clp in bacteria. It also provided a new thought and the method to increase production of HSAF. The production of HSAF is effectively increased and it is a key part of industrialization.

Our results suggest that LesR exerts its regulation through HSAF biosynthesis to control Clp protein levels (Fig. 2); however, this finding is not consistent with that of the mRNA results, where we found that the overexpressed *lesR* did not have a significant influence on the *clp* transcript, which was determined by RNA-Seq and RT-PCR (Fig. 1 and Fig. 4a). These findings collectively raise the possibility that LesR may affect the Clp protein level or its stability in a post-transcriptional manner. In marked contrast to the effect of LesR on the Clp levels, inactivation of DSF signaling did not seem to alter the Clp protein level (Additional file 1: Figure S1). These results, along with our recent report [17], suggest that although Clp acts as a downstream regulator in both the DSF and LesR regulatory pathways, each pathway appears to use different Clp-dependent mechanism(s) to regulate HSAF biosynthesis in *L. enzymogenes*.

In contrast to the regulatory effect on HSAF biosynthesis, LesR performs its regulation of cell aggregation via a Clp-dependent, but not DSF-dependent, manner because overexpression of *lesR* in the *rpfF* mutant (the DSF-disruption mutant) still caused significant cell aggregation similar to that in the wild-type OH11 [18]. Moreover, Clp is also involved in the regulation of several important biocontrol characteristics in both LesR- and DSF-independent manners. Two representative examples are the extracellular chitinase production and type IV pilus-dependent twitching motility because these Clp-controlled phenotypes are not influenced by either the disruption of DSF signaling or the overexpression of *lesR* in *L. enzymogenes* [14, 15, 18]. Collectively, the above findings suggest that Clp functions as a diverse regulatory player in *L. enzymogenes*. First, it seems to be at the intersection of the LesR and DSF regulatory pathways in performing their respective functions on HSAF biosynthesis. Second, Clp switches to mediate LesR-controlled cell aggregation in a DSF-independent manner. Third, Clp has evolved to acquire the ability to perform its unique regulation unrelated to both the LesR and the DSF regulatory pathways.

Finally, it is important to note that in addition to Clp, the LuxR solos are also widely distributed in diverse phytopathogenic *Xanthomonas* species, such as XccR of *Xcc*, OryR of *X. oryzae* and XagR of *X. axonopodis* [21–23]. In these phytopathogenic bacteria, although the role of Clp or the LuxR solo in virulence has been reported, their genetic relationships are poorly understood. In this regard, the genetic relationship of LesR and Clp presented in this work should be followed by respective studies in those pathogenic *Xanthomonas* species.

## Conclusions

LesR is a LuxR solo that controls Clp expression to modulate HSAF biosynthesis as well as cell aggregation. This finding was subsequently validated by comparative transcriptome analysis, which found that the regulatory action of LesR largely overlaps with that of Clp. Therefore, in addition to the well-known DSF signaling system, the present study revealed a new upstream regulatory factor for Clp in *L. enzymogenes*. The key factor was important for the production of HSAF. The strains of high yield HSAF will be constructed by deletion of negative regulators or overexpression of positive regulators by genetic engineering.

## Methods

### Bacterial strains, culture media and growth conditions

The bacterial strains and plasmids used in this study are listed in Table 1. *Escherichia coli* strains were used for plasmid construction and were grown at 37 °C in Luria-Bertani (LB) broth in the presence or absence of 100 μg/ml ampicillin (Amp) or 25 μg/ml gentamicin (Gm). *Lysobacter enzymogenes* OH11 (CGMCC No. 1978) and its derivative strains were grown in LB broth or 1/10 TSB (Trypic Soy Broth, Sigma) at 28 °C with shaking at 200 rpm. When required, antibiotics were added to the medium at a final concentration of 100 μg/ml kanamycin (km) and/or 150 μg/ml Gm.

**Table 1:** Bacterial strains and plasmids used in this study

Strains and plasmids	Characteristics^a^	Source
Strains		
*Lysobacter enzymogenes*		
OH11	Wild-type strain OH11, Km^R^	[24] CGMCC no. 1978
OH11 (*lesR*)	OH11 harboring plasmid pBBR-*lesR*, Km^R^, Gm^R^	[18]
OH11 (pBBR)	OH11 harboring plasmid pBBR1-MCS5, Km^R^, Gm^R^	[18]
OH11 (*lesR*-*clp*)	OH11 harboring plasmid pBBR-*lesR*-*clp*, Km^R^, Gm^R^	This study
OH11 (*clp*)	OH11 harboring plasmid pBBR-*clp*, Km^R^, Gm^R^	This study
Δ*clp*	*clp* in-frame deletion mutant of strain OH11, Km^R^	[15]
Δ*clp* (*lesR*)	Mutant Δ*clp* harboring plasmid pBBR1-*lesR*, Km^R^, Gm^R^	This study
Δ*clp* (pBBR)	Mutant Δ*clp* harboring plasmid pBBR1-MCS5, Km^R^, Gm^R^	[15]
Δ*rpfF*	The *rpfF* in-frame deletion mutant of strain OH11, Km^R^	[14]
Δ*rpfF* (*rpfF*)	Δ*rpfF* harboring plasmid pBBR1-*rpfF*, Km^R^, Gm^R^	[14]
Δ*rpfF* (pBBR)	Δ*rpfF* harboring plasmid pBBR1-MCS5, Km^R^, Gm^R^	[14]
*Escherichia coli*		
DH5a	F^−^, φ80*dlacZ*∆M15, ∆(*lacZYA*-*argF*) *U169, deoR, recA1, endA1, hsdR17*(*r*_*k*_^−^ *, m*_*k*_^+^)*, phoA, supE44, λ* ^−^ *, thi*-*1, gyrA96*	[14]
Plasmids		
pMD19-T	ColE1 origin, T Simple vector, Amp^R^	TaKaRa, Shanghai, China
pBBR1-MCS5	Broad host range cloning vector, *lacZ*, Gm^R^	[25]
pBBR-*clp*	pBBR1-MCS5 with 1.2 kb fragment including *clp,* Gm^R^	[15]
pBBR-*lesR*	pBBR1-MCS5 with 1.5 kb fragment including *lesR,* Gm^R^	[18]
pBBR-*lesR*-*clp*	pBBR-*lesR* harboring a 1.25-kp DNA fragment, which contained the coding region of *clp* and its native promoter region, Gm^R^	This study

^a^Km^R^, Gm^R^ and Amp^R^ indicate resistance to kanamycin, gentamicin and ampicillin, respectively

### Introduction of *clp* into the *lesR* overexpression strain

To introduce *clp* into the *lesR* overexpression strain, a 1250-bp DNA fragment including the coding region of *clp* and its native promoter region was amplified by PCR with the corresponding primer pairs (Additional file 1: Table S1). This DNA fragment was cloned into pBBR-*lesR*, where *lesR* under the control of its native promoter was cloned into the broad-host vector pBBR1-MCS5 (Table 1). The final construct, pBBR-*lesR*-*clp*, was transformed into wild-type OH11, generating the strain OH11(*lesR*-*clp*) (Table 1). The overexpression of *clp* in the corresponding transformed strain was validated by RT-PCR (reverse transcription PCR), which is presented in detail below. The strain OH11(*lesR*-*clp*) was used for further study.

### Extraction and HPLC analysis of HSAF

The wild-type OH11 of *L. enzymogenes* and its derivatives were cultivated in 1/10 TSB broth for 2 days and adjusted to the same cell density (OD_600_ = 1.8). Then, HSAF was extracted from these various *L. enzymogenes* cultures and detected by HPLC (High-Performance Liquid Chromatography) as described previously [4, 14, 18]. In the HPLC analysis, the standard HSAF [4, 14] was used as a control. Three replications were used for each treatment, and the experiment was repeated three times.

### Cell aggregation assay

The cell aggregation assay was performed according to our earlier report [18]. In brief, the wild-type strain OH11 and its derivative strains were pre-incubated in LB broth at 28 °C with shaking at 200 rpm until the culture reached an OD_600_ of 2.0. Then, these cultures were vertically placed without movement in a tube support. After 12 h, the aggregated cells at the bottom of each tube were observed and photographed. Three technical replicates for each treatment were used, and the experiment was carried out three times.

### Western blot analysis

The western blot analysis was performed according to a published laboratory protocol with a minor modification [26]. In brief, the designated control strain (wild-type OH11 containing the empty vector pBBR1-MCS5) and the *lesR* overexpression strain were pre-incubated in LB broth at 28 °C with shaking at 200 rpm until the culture reached an OD_600_ of 2.0. The cells were harvested by centrifugation at 3381×*g* and frozen at − 80 °C. The cells were lysed by 6 × Loading buffer (60 mM Tris–Cl pH 6.8, 600 mM DTT, 4% SDS, 20% Glycerol, 0.2% Bromophenol Blue). Soluble proteins were harvested from these bacterial cells, further separated by SDS-PAGE and transferred onto a polyvinylidene difluoride (PVDF) membrane using a semi-dry blot machine (Bio-RAD, USA). The membranes were probed using a polyclonal antibody specific for *Xcc* Clp with a dilution of 1:5000 [26], followed by detection with an HRP-conjugated anti-rabbit secondary antibody (No. M21002, Abmart, China).

### RT-PCR assay

The wild-type OH11 and its derivatives were grown in 1/10 TSB broth. The cells were collected at an OD_600_ of 1.0. Then, the total RNA was extracted from the cells of each strain using a kit with a code of R6950-01 from OMEGA (China). Next, RT-PCR, including cDNA synthesis using the PrimerScript RT Reagent Kit with gDNA Eraser (number RR047A; TaKaRa, China) and PCR amplification, was performed as described previously [14, 18]. The *16* *s rRNA* gene was used as an internal control in this study [14, 18]. The primer sequences used in this assay are listed in Additional file 1: Table S1.

### RNA-seq analysis

RNA-Seq-based transcriptome profiling was performed by the Beijing Genomics Institute (Shenzhen, China) using the Illumina HiSeq™ 2000 platform. In brief, the experiment was performed as follows: the control strain (wild-type OH11 containing an empty vector) and the *lesR* overexpression strain were grown in 1/10 TSB until the cultures reached an OD_600_ of 1.0. The cells of each strain were collected by centrifugation (10,000×*g* at 4 °C for 3 min) and were used for RNA extraction according to standard protocols. After the total RNA extraction and DNase I treatment, rRNAs were removed from the total RNA. The mRNA was mixed with fragmentation buffer for fragmentation into short fragments. Then cDNA was synthesized using the mRNA fragments as templates. The short fragments were purified and used for end repair and single nucleotide A (adenine) addition and connected with adapters. The suitable fragments were selected for PCR amplification through agarose gel electrophoresis. During the quality control steps, an Agilent 2100 Bioanaylzer and an ABI StepOnePlus Real-Time PCR System were used for quantification of the sample library. Finally, the library was sequenced using an Illumina HiSeq™ 2000. The RPKM (Reads Per Kilobase per Million mapped reads) was used to identify significantly differentially expressed genes between the control strain and the *lesR* overexpression strain as described previously [27]. In this way, the differentially expressed genes with a selection threshold of a false discovery rate of FDR ≤ 0.001 and fold change ≥ 2.0 were selected for further study.

## Additional file

**Additional file 1.** **Table S1.** Primers used in this study. **Table S2.** Genes controlled by LesR in *Lysobacter enzymogenes.* **Figure S1.** *Le*DSF signaling did not control the Clp protein level.

## Abbreviations

DSF: diffusible signal factor
HSAF: heat-stable antifungal factor
*Xcc*: *Xanthomonas campestris* pv. *campestris*
Clp: cAMP-receptor like protein
HTH: helix-turn-helix
EPS: extracellular polysaccharide
RT-PCR: reverse transcription-PCR
PVDF: polyvinylidene difluoride
RPKM: Reads Per Kilobase per Million mapped reads
LB: Luria–Bertani
TSB: Trypic Soy Broth
Km: kanamycin
Gm: gentamicin
PCR: polymerase chain reaction
HPLC: High-Performance Liquid Chromatography

## References

[1] Christensen P, Cook FD (1978). *Lysobacter*, a new genus of nonfruiting, gliding bacteria with high base ratio. Int J Syst Bacteriol.

[2] Kobayashi DY, Reedy RM, Palumbo JD, Zhou JM, Yuen GY (2005). A *clp* gene homologue belonging to the *Crp* gene family globally regulates lytic enzyme production, antimicrobial activity and biological control activity expressed by *Lysobacter enzymogenes* strain C3. Appl Environ Microbiol.

[3] Li SJ, Du LC, Yuen GY, Harris SD (2006). Distinct ceramide synthases regulate polarized growth in the filamentous fungus *Aspergillus nidulans*. Mol Biol Cell.

[4] Yu FG, Zaleta-Rivera K, Zhu XC, Huffman J, Millet JC, Harris SD, Yuen GY, Li XC, Du LC (2007). Structure and biosynthesis of heat-stable antifungal factor (HSAF), a broad-spectrum antimycotic with a novel mode of action. Antimicrob Agents Chemother.

[5] Xu LX, Wu P, Wright SJ, Du LC, Wei XY (2015). Bioactive polycyclic tetramate macrolactams from *Lysobacter enzymogenes* and their absolute configurations by theoretical ECD calculations. J Nat Prod.

[6] He YW, Ng YJ, Xu M, Lin K, Wang LH, Dong YH, Zhang LH (2007). *Xanthomonas campestris* cell-cell communication involves a putative nucleotide receptor protein Clp and a hierarchical signaling network. Mol Microbiol.

[7] Chin KH, Lee YC, Tu ZL, Chen CH, Tseng YH, Yang JM, Ryan RP, McCarthy Y, Dow JM, Wang AH, Chou SH (2010). The cAMP receptor-like protein CLP is a novel c-di-GMP receptor linking cell-cell signaling to virulence gene expression in *Xanthomonas campestris*. J Mol Biol.

[8] Tao F, He YW, Wu DH, Swarup S, Zhang LH (2010). The cyclic nucleotide monophosphate domain of *Xanthomonas campestris* global regulator Clp defines a new class of cyclic di-GMP effectors. J Bacteriol.

[9] Barber CE, Tang JL, Feng JX, Pan MQ, Wilson TJ, Slater H, Dow JM, Williams P, Daniels MJ (1997). A novel regulatory system required for pathogenicity of *Xanthomonas campestris* is mediated by a small diffusible signal molecule. Mol Microbiol.

[10] He YW, Xu M, Lin K, Ng YJ, Wen CM, Wang LH, Liu ZD, Zhang HB, Dong YH, Dow JM, Zhang LH (2006). Genome scale analysis of diffusible signal factor regulon in *Xanthomonas campestris* pv. *campestris*: identification of novel cell-cell communication-dependent genes and functions. Mol Microbiol.

[11] He YW, Wang C, Zhou L, Song HW, Dow JM, Zhang LH (2006). Dual signaling functions of the hybrid sensor kinase RpfC of *Xanthomonas campestris* involve either phosphorelay or receiver domain-protein interaction. J Biol Chem.

[12] He YW, Zhang LH (2008). Quorum sensing and virulence regulation in *Xanthomonas campestris*. FEMS Microbiol Rev.

[13] Ryan RP, Fouhy Y, Lucey JF, Crossman LC, Spiro S, He YW, Zhang LH, Heeb S, Cámara M, Williams P, Dow JM (2006). Cell–cell signaling in *Xanthomonas campestris* involves an HD-GYP domain protein that functions in cyclic di-GMP turnover. Proc Nat Acad Sci.

[14] Qian GL, Wang YL, Liu YR, Xu FF, He YW, Du LC, Venturi V, Fan JQ, Hu BS, Liu FQ (2013). *Lysobacter enzymogenes* uses two distinct cell-cell signaling systems for differential regulation of secondary-metabolite biosynthesis and colony morphology. Appl Environ Microbiol.

[15] Wang YS, Zhao YX, Zhang J, Zhao YY, Shen Y, Su ZH, Xu GG, Du LC, Huffman JM, Venturi V, Qian GL, Liu FQ (2014). Transcriptomic analysis reveals new regulatory roles of Clp singaling in secondary-metabolite biosysnthesis and surface motility in *Lysobacter enzymogenes* OH11. Appl Microbiol Biotechnol.

[16] Han Y, Wang Y, Tombosa S, Wright S, Huffman J, Yuen GY, Qian GL, Liu FQ, Shen YM, Du LC (2015). Identification of a small molecule signaling factor that regulates the biosynthesis of the antifungal polycyclic tetramate macrolactam HSAF in *Lysobacter enzymogenes*. Appl Microbiol Biotechnol.

[17] Xu GG, Shi XF, Wang RP, Xu HY, Du LC, Chou SH, Liu HX, Liu YZ, Qian GL, Liu FQ (2016). Insights into the distinct cooperation between the transcription factor Clp and LeDSF signaling in the regulation of antifungal factors in *Lysobacter enzymogenes* OH11. Biol Control.

[18] Qian GL, Xu FF, Venturi V, Du LC, Liu FQ (2014). Roles of a solo LuxR in the biological control agent *Lysobacter enzymogenes* strain OH11. Phytopathology.

[19] Subramoni S, Venturi V (2009). LuxR-family ‘solos’: bachelor sensors/regulators of signalling molecules. Microbiology.

[20] Gonzalez JF, Venturi V (2013). A novel widespread interkingdom signaling circuit. Trends Plant Sci.

[21] Ferluga S, Bigirimana J, Höfte M, Venturi V (2007). A, LuxR homologue of Xanthomonas oryzae pv. oryzae is required for optimal rice virulence. Mol. Plant Pathol.

[22] Zhang L, Jia Y, Wang L, Fang R (2007). A proline iminopeptidase gene upregulated in planta by a LuxR homologue is essential for pathogenicity of *Xanthomonas campestris* pv. *campestris*. Mol Microbiol.

[23] Chatnaparat T, Prathuangwong S, Ionescu M, Lindow SE (2012). XagR, a LuxR homolog, contributes to the virulence of *Xanthomonas axonopodis* pv. *glycines* to soybean. Mol Plant Microbe Interact.

[24] Qian GL, Hu BS, Jiang YH, Liu FQ (2009). Identification and characterization of *Lysobacter enzymogenes* as a biological control agent against some fungal pathogens. Agr Sci China..

[25] Kovach ME, Elzer PH, Hill DS, Robertson GT, Farris MA, Roop RM, Peterson KM (1995). Four new derivatives of the broad-host-range cloning vector pBBR1-MCS, carrying different antibiotic-resistance cassettes. Gene.

[26] Xu HY, Chen HF, Shen YM, Du LC, Chou SH, Liu HX, Qian GL, Liu FQ (2016). Direct regulation of extracellular chitinase production by the transcription factor LeClp in *Lysobacter enzymogenes* OH11. Phytopathology..

[27] Mortazavi A, Williams BA, McCue K, Schaeffer L, Wold B (2008). Mapping and quantifying mammalian transcriptomes by RNA-Seq. Nat Methods.

