# Supplementary material for: LesR is a novel upstream regulator that controls downstream Clp expression to modulate antibiotic HSAF biosynthesis and cell aggregation in Lysobacter enzymogenes OH11
Source: Microb Cell Fact. 2017 Nov 14;16:202. doi: 10.1186/s12934-017-0818-2 (PMC5686890; doi:10.1186/s12934-017-0818-2)
Supplement: Supplementary file 1 — Additional file 1. Table S1. Primers used in this study. Table S2. Genes controlled by LesR in Lysobacter enzymogenes. Figure S1. LeDSF signaling did not control the Clp protein level. [file 12934_2017_818_MOESM1_ESM.doc]

**LesR is a novel upstream regulator that controls downstream Clp expression to modulate antibiotic HSAF biosynthesis and cell aggregation in** ***Lysobacter enzymogenes* OH11**

Huiyong Xu^1^, Ruping Wang^2^, Yangyang Zhao^1^, Zhengqing Fu^3^, Guoliang Qian^2^, Fengquan Liu*^1^

^1^ Institute of Plant Protection, Jiangsu Academy of Agricultural Sciences, Nanjing 210014, P.R. China

^2^ College of Plant Protection, Nanjing Agricultural University, Nanjing 210095, China/Key Laboratory of Integrated Management of Crop Diseases and Pests (Nanjing Agricultural University), Ministry of Education

^3^ Department of Biological Sciences, University of South Carolina, Columbia, SC 29208, USA.

**Email addresses**

Huiyong Xu: xuhy0921@163.com (submitting author)

Ruping Wang: 2013102043@njau.edu.cn

Yangyang Zhao: yyzhao2016@163.com

Zhengqing Fu: zfu@mailbox.sc.edu

Guoliang Qian: glqian@njau.edu.cn

Fengquan Liu^*^: fqliu20011@sina.com (^*^corresponding author)

**Postal address:** Institute of Plant Protection, Jiangsu Academy of Agricultural Sciences, Nanjing 210014, P.R. China; Tel: +86-25-84390277. Fax: +86-25-84390277

**
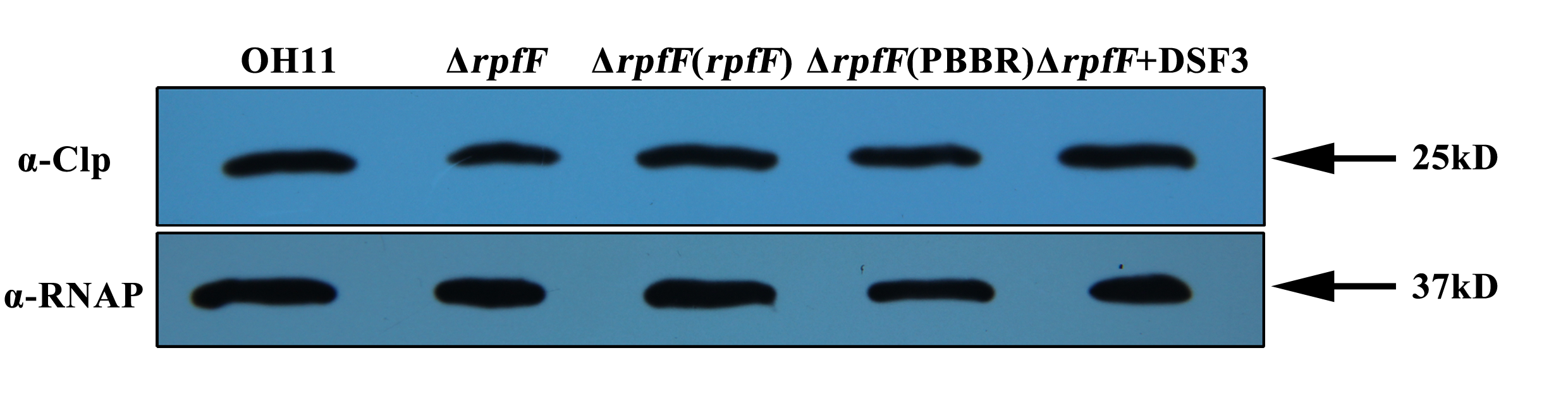
**

**Figure S1. *Le*DSF signaling did not control the Clp protein level.** A 25-kDa band corresponding to the size of Clp was detected by the polyclonal antibody α-Clp in various *L. enzymogenes* strains. The RNA polymerase α-subunit (37 kDa) was used as the internal control for loading, which was detected by the specific antibody α-RNAP. OH11, the wild-type strain; Δ*rpfF*, the *rpfF* deletion mutant; Δ*rpfF* (*rpfF*), the complemented strain of Δ*rpfF*; Δ*rpfF* (pBBR), Δ*rpfF* containing an empty vector; and Δ*rpfF* +DSF3, Δ*rpfF* treated with 10 μM *Le*DSF3.

**Table S1. Primers used in this study.**

| **Primer sequence ^a^** | **Purpose** | **Source** |
| --- | --- | --- |
| 5'-ACGGTCGCAAGACTGAAACT-3'  5'-AAGGCACCAATCCATCTCTG-3' | RT-PCR, an internal control (*16S rRNA*) | [18] |
| 5'-AGTGGTCGTTCACCACCTTC-3'  5'-TCATCGAGATCTCCCACGAG-3' | RT-PCR for monitoring the transcription of *lesR* | [18] |
| 5'-GGCTCGGTCAGCATCATTAC-3'  5'-GATGGTCGCTCTCGATGAA-3' | RT-PCR for monitoring the transcription of *clp* | [18] |
| 5'-CCCAAGCTTATCTGGGTGAGGCGGTCGGC-3' (*Hin*dIII);  5'-CCCGGTACCGCAGAAGGTAATGGCGCGGT-3' (*Kpn*I) | To amplify a 1250-bp fragment containing the coding region of *clp* and its native promoter region | This study |

^a^The restriction digestion enzyme site is underlined.

***Table S2. Genes controlled by LesR in Lysobacter enzymogenes.***

| **geneID ^a^** | **OH11(pBBR)-**  **RPKM** | **OH11(*lesR*)-**  **RPKM** | **log2 Ratio** | **P-value** | **FDR** | **Function annotation** |
| --- | --- | --- | --- | --- | --- | --- |
| OH11GL000027 | 90.38497 | 36.94789 | -1.29059 | 3.77E-11 | 1.36E-10 | Function unknown |
| OH11GL000070 | 56.20751 | 115.3976 | 1.037779 | 4.40E-12 | 1.78E-11 | Secondary metabolites biosynthesis, transport and catabolism |
| OH11GL000079 | 21.23185 | 55.17833 | 1.377872 | 0 | 0 | Function unknown |
| OH11GL000085 | 110.7959 | 52.8723 | -1.06732 | 2.52E-11 | 9.23E-11 | Function unknown |
| OH11GL000087 | 34.10034 | 90.76388 | 1.412332 | 2.00E-12 | 8.64E-12 | Function unknown |
| OH11GL000098 | 10.59326 | 4.577303 | -1.21058 | 2.03E-15 | 1.13E-14 | Cell wall/membrane/envelope biogenesis |
| OH11GL000123 | 24.64125 | 9.012797 | -1.45103 | 3.27E-35 | 2.46E-34 | Function unknown |
| OH11GL000153 | 84.76596 | 171.983 | 1.020709 | 1.86E-13 | 9.40E-13 | Function unknown |
| OH11GL000188 | 57.62218 | 243.4782 | 2.079097 | 0 | 0 | Function unknown |
| OH11GL000192 | 33.33346 | 175.9254 | 2.39992 | 2.55E-14 | 1.39E-13 | Function unknown |
| OH11GL000193 | 8.088267 | 19.14822 | 1.243308 | 0.000174 | 0.000388 | Function unknown |
| OH11GL000195 | 107.7819 | 49.20882 | -1.13113 | 2.02E-37 | 1.54E-36 | Function unknown |
| OH11GL000200 | 216.003 | 919.425 | 2.089681 | 2.37E-11 | 8.69E-11 | Posttranslational modification,protein turnover,chaperones |
| OH11GL000217 | 22.58073 | 52.92322 | 1.228808 | 2.61E-09 | 8.32E-09 | Function unknown |
| OH11GL000227 | 25.55002 | 52.25942 | 1.032367 | 1.02E-12 | 4.61E-12 | Posttranslational modification,protein turnover,chaperones |
| OH11GL000229 | 35.62514 | 94.7142 | 1.410685 | 0 | 0 | Function unknown |
| OH11GL000230 | 335.1865 | 912.0984 | 1.444225 | 0 | 0 | Function unknown |
| OH11GL000239 | 183.5234 | 379.6744 | 1.048798 | 4.60E-12 | 1.86E-11 | Cell motility |
| OH11GL000240 | 144.1111 | 299.5617 | 1.055671 | 0 | 0 | Cell motility |
| OH11GL000241 | 81.20837 | 229.3057 | 1.497572 | 1.54E-12 | 6.79E-12 | Cell motility |
| OH11GL000268 | 58.88453 | 15.44799 | -1.93047 | 8.14E-107 | 8.24E-106 | Defense mechanisms |
| OH11GL000269 | 156.9859 | 36.40715 | -2.10834 | 1.06E-296 | 1.31E-295 | Defense mechanisms |
| OH11GL000270 | 139.2213 | 32.6478 | -2.09232 | 5.59E-295 | 6.87E-294 | Function unknown |
| OH11GL000271 | 342.8274 | 59.17855 | -2.53434 | 0 | 0 | Transcription |
| OH11GL000328 | 308.0521 | 691.4114 | 1.16637 | 0 | 0 | Defense mechanisms |
| OH11GL000345 | 376.6015 | 177.6383 | -1.0841 | 6.93E-98 | 6.87E-97 | Function unknown |
| OH11GL000369 | 33.86018 | 307.9922 | 3.185232 | 0 | 0 | Carbohydrate transport and metabolism |
| OH11GL000371 | 52.14467 | 415.9991 | 2.995989 | 2.54E-12 | 1.08E-11 | Energy production and conversion |
| OH11GL000372 | 20.07489 | 236.6472 | 3.559274 | 3.14E-13 | 1.53E-12 | Inorganic ion transport and metabolism |
| OH11GL000373 | 33.95453 | 101.54 | 1.580372 | 1.44E-12 | 6.38E-12 | Signal transduction mechanisms |
| OH11GL000378 | 6.573731 | 19.71275 | 1.584345 | 0 | 0 | Function unknown |
| OH11GL000458 | 380.2736 | 20.78603 | -4.19335 | 0 | 0 | Function unknown |
| OH11GL000459 | 31.80285 | 3.602124 | -3.14224 | 3.16E-84 | 3.05E-83 | Function unknown |
| OH11GL000469 | 46.19583 | 122.6596 | 1.408826 | 0 | 0 | Function unknown |
| OH11GL000471 | 63.35327 | 141.463 | 1.158934 | 0 | 0 | Amino acid transport and metabolism |
| OH11GL000472 | 13.33339 | 42.05233 | 1.657143 | 1.88E-08 | 5.72E-08 | Function unknown |
| OH11GL000473 | 313.819 | 644.6338 | 1.038547 | 0 | 0 | Function unknown |
| OH11GL000474 | 649.0222 | 1479.775 | 1.189038 | 2.07E-12 | 8.95E-12 | Function unknown |
| OH11GL000478 | 6.928866 | 14.9227 | 1.106818 | 6.88E-15 | 3.80E-14 | Function unknown |
| OH11GL000524 | 33.96752 | 11.32243 | -1.58497 | 3.22E-13 | 1.57E-12 | Function unknown |
| OH11GL000625 | 331.2513 | 145.8329 | -1.18361 | 5.18E-111 | 5.30E-110 | General function prediction only |
| OH11GL000631 | 156.558 | 69.8237 | -1.16491 | 1.35E-67 | 1.22E-66 | Function unknown |
| OH11GL000632 | 3400.582 | 1209.75 | -1.49107 | 0 | 0 | Function unknown |
| OH11GL000660 | 5.60347 | 17.06173 | 1.606371 | 1.83E-08 | 5.58E-08 | Function unknown |
| OH11GL000714 | 71.13515 | 149.2251 | 1.068856 | 2.46E-13 | 1.22E-12 | Cell wall/membrane/envelope biogenesis |
| OH11GL000857 | 19.03943 | 42.75964 | 1.16726 | 4.42E-13 | 2.11E-12 | Function unknown |
| OH11GL000887 | 8.227073 | 74.19784 | 3.172926 | 1.27E-12 | 5.70E-12 | Function unknown |
| OH11GL000888 | 23.46992 | 91.23534 | 1.95878 | 3.38E-12 | 1.41E-11 | Function unknown |
| OH11GL000890 | 17.27238 | 67.16029 | 1.959142 | 0 | 0 | Function unknown |
| OH11GL000891 | 4.500018 | 14.35181 | 1.67323 | 1.50E-09 | 4.85E-09 | Function unknown |
| OH11GL000906 | 6.826268 | 15.88352 | 1.218362 | 4.47E-12 | 1.81E-11 | Function unknown |
| OH11GL000926 | 216.2887 | 3628.461 | 4.068327 | 0 | 0 | Function unknown |
| OH11GL000927 | 9.002147 | 66.80431 | 2.8916 | 0 | 0 | Defense mechanisms |
| OH11GL000962 | 19.74082 | 89.5316 | 2.181215 | 0 | 0 | Function unknown |
| OH11GL000963 | 70.26094 | 145.1417 | 1.046667 | 2.34E-12 | 1.00E-11 | Function unknown |
| OH11GL000964 | 28.46332 | 57.19378 | 1.006755 | 0 | 0 | Defense mechanisms |
| OH11GL000982 | 26.65027 | 90.18581 | 1.75875 | 4.53E-13 | 2.16E-12 | Function unknown |
| OH11GL001026 | 43.75017 | 19.72847 | -1.14901 | 8.43E-06 | 2.15E-05 | Function unknown |
| OH11GL001035 | 16.87905 | 59.71318 | 1.822816 | 7.77E-15 | 4.29E-14 | Function unknown |
| OH11GL001036 | 18.3545 | 217.3428 | 3.565767 | 1.33E-13 | 6.84E-13 | Function unknown |
| OH11GL001037 | 23.99563 | 269.1351 | 3.487487 | 3.61E-13 | 1.74E-12 | Function unknown |
| OH11GL001048 | 2.51939 | 21.26393 | 3.077262 | 1.93E-08 | 5.86E-08 | Function unknown |
| OH11GL001066 | 443.9338 | 139.057 | -1.67467 | 2.21E-185 | 2.52E-184 | Function unknown |
| OH11GL001085 | 68.58135 | 170.326 | 1.312411 | 6.82E-14 | 3.60E-13 | Function unknown |
| OH11GL001106 | 3.216032 | 8.680528 | 1.432501 | 2.72E-12 | 1.16E-11 | Function unknown |
| OH11GL001111 | 2.828065 | 1.099795 | -1.36258 | 4.02E-05 | 9.59E-05 | General function prediction only |
| OH11GL001113 | 57.56153 | 18.16525 | -1.66392 | 6.61E-86 | 6.41E-85 | Amino acid transport and metabolism |
| OH11GL001114 | 132.4286 | 37.93917 | -1.80345 | 6.95E-187 | 7.93E-186 | Amino acid transport and metabolism |
| OH11GL001126 | 14.6068 | 43.89031 | 1.587262 | 0 | 0 | Cell motility |
| OH11GL001127 | 17.03194 | 46.75726 | 1.456948 | 3.80E-13 | 1.82E-12 | Cell motility |
| OH11GL001128 | 14.29583 | 32.57032 | 1.187963 | 6.82E-14 | 3.60E-13 | Cell motility |
| OH11GL001129 | 8.875565 | 39.70697 | 2.161481 | 3.29E-14 | 1.77E-13 | Cell motility |
| OH11GL001130 | 1.824825 | 14.00289 | 2.939895 | 6.13E-11 | 2.17E-10 | Cell motility |
| OH11GL001131 | 9.144005 | 75.0511 | 3.036975 | 0 | 0 | Cell motility |
| OH11GL001133 | 12.12505 | 81.94418 | 2.756651 | 0 | 0 | Cell motility |
| OH11GL001134 | 10.21855 | 82.0745 | 3.005744 | 1.39E-13 | 7.14E-13 | Cell motility |
| OH11GL001135 | 4.303296 | 55.78306 | 3.696313 | 2.71E-11 | 9.91E-11 | Function unknown |
| OH11GL001136 | 1.94806 | 19.84121 | 3.34839 | 4.34E-08 | 1.29E-07 | Cell motility |
| OH11GL001137 | 2.380962 | 25.29754 | 3.409381 | 2.16E-07 | 6.12E-07 | Cell motility |
| OH11GL001138 | 4.278367 | 34.72211 | 3.020722 | 1.69E-13 | 8.59E-13 | Cell motility |
| OH11GL001139 | 4.371602 | 18.02514 | 2.043776 | 9.35E-14 | 4.87E-13 | Function unknown |
| OH11GL001140 | 2.564113 | 39.7672 | 3.955048 | 2.58E-12 | 1.10E-11 | Function unknown |
| OH11GL001141 | 5.191894 | 35.30996 | 2.765742 | 0 | 0 | Cell motility |
| OH11GL001142 | 2.50001 | 17.25255 | 2.786804 | 1.65E-09 | 5.33E-09 | Function unknown |
| OH11GL001143 | 0.643779 | 6.705987 | 3.380811 | 4.78E-06 | 1.24E-05 | Function unknown |
| OH11GL001144 | 21.92038 | 132.724 | 2.598085 | 0 | 0 | Cell motility |
| OH11GL001145 | 25.41677 | 197.668 | 2.959227 | 6.52E-13 | 3.02E-12 | Cell motility |
| OH11GL001146 | 2.50001 | 18.84915 | 2.914493 | 2.10E-13 | 1.06E-12 | Function unknown |
| OH11GL001147 | 8.114067 | 55.73812 | 2.780167 | 2.93E-14 | 1.59E-13 | Cell motility |
| OH11GL001148 | 2.293587 | 9.556544 | 2.058883 | 1.92E-06 | 5.14E-06 | Function unknown |
| OH11GL001149 | 8.58782 | 41.34847 | 2.26747 | 0 | 0 | Cell motility |
| OH11GL001150 | 5.413407 | 26.52004 | 2.292474 | 0 | 0 | Transcription |
| OH11GL001151 | 6.338053 | 29.91318 | 2.23867 | 0 | 0 | Function unknown |
| OH11GL001152 | 4.347843 | 16.52236 | 1.926048 | 3.35E-14 | 1.80E-13 | Cell wall/membrane/envelope biogenesis |
| OH11GL001154 | 4.250017 | 9.114554 | 1.100704 | 5.10E-05 | 0.000121 | Function unknown |
| OH11GL001156 | 104.5143 | 214.8431 | 1.039583 | 0 | 0 | Function unknown |
| OH11GL001157 | 25.72014 | 264.5384 | 3.362507 | 4.46E-12 | 1.81E-11 | Function unknown |
| OH11GL001200 | 17629 | 8046.105 | -1.13159 | 0 | 0 | Transcription |
| OH11GL001221 | 56.06531 | 126.7665 | 1.176994 | 1.53E-13 | 7.83E-13 | Function unknown |
| OH11GL001244 | 41.72766 | 18.75501 | -1.15373 | 1.43E-40 | 1.14E-39 | Carbohydrate transport and metabolism |
| OH11GL001266 | 7.608726 | 3.187054 | -1.25543 | 1.53E-05 | 3.80E-05 | Posttranslational modification, protein turnover, chaperones |
| OH11GL001290 | 21.54264 | 44.60314 | 1.04995 | 0 | 0 | Function unknown |
| OH11GL001293 | 1115.964 | 517.8848 | -1.10759 | 0 | 0 | Nucleotide transport and metabolism |
| OH11GL001294 | 58.4713 | 148.8962 | 1.348506 | 2.10E-12 | 9.06E-12 | Secondary metabolites biosynthesis, transport and catabolism |
| OH11GL001309 | 266.0427 | 128.0378 | -1.05509 | 3.87E-64 | 3.45E-63 | Function unknown |
| OH11GL001367 | 228.9508 | 589.0833 | 1.363434 | 0 | 0 | Inorganic ion transport and metabolism |
| OH11GL001385 | 13.10769 | 51.78176 | 1.98203 | 2.29E-14 | 1.24E-13 | General function prediction only |
| OH11GL001409 | 3.947384 | 16.7519 | 2.085356 | 4.47E-06 | 1.17E-05 | Function unknown |
| OH11GL001415 | 218.8068 | 91.41762 | -1.25911 | 3.06E-192 | 3.50E-191 | Energy production and conversion |
| OH11GL001416 | 652.1335 | 234.1757 | -1.47758 | 0 | 0 | Energy production and conversion |
| OH11GL001418 | 515.9037 | 98.70187 | -2.38595 | 0 | 0 | Coenzyme transport and metabolism |
| OH11GL001458 | 9.090945 | 18.81307 | 1.049233 | 1.99E-13 | 1.00E-12 | Function unknown |
| OH11GL001488 | 2633.02 | 1050.597 | -1.32551 | 0 | 0 | Function unknown |
| OH11GL001531 | 40.17184 | 332.0646 | 3.047207 | 0 | 0 | Cell wall/membrane/envelope biogenesis |
| OH11GL001566 | 1.701577 | 3.772166 | 1.148521 | 0.000202 | 0.000448 | Cell wall/membrane/envelope biogenesis |
| OH11GL001567 | 18.00007 | 38.65728 | 1.102738 | 2.82E-07 | 7.92E-07 | Function unknown |
| OH11GL001568 | 0.644031 | 5.366884 | 3.058883 | 9.71E-08 | 2.82E-07 | Carbohydrate transport and metabolism |
| OH11GL001655 | 37.50015 | 77.25669 | 1.042764 | 4.91E-10 | 1.63E-09 | Function unknown |
| OH11GL001681 | 6.458359 | 13.85268 | 1.100925 | 1.59E-13 | 8.10E-13 | Function unknown |
| OH11GL001701 | 67.23629 | 165.0918 | 1.295956 | 1.87E-12 | 8.11E-12 | Function unknown |
| OH11GL001702 | 86.56284 | 249.9992 | 1.530104 | 0 | 0 | Function unknown |
| OH11GL001715 | 11.19796 | 35.44549 | 1.662366 | 8.86E-14 | 4.64E-13 | General function prediction only |
| OH11GL001734 | 8.373239 | 33.9745 | 2.020595 | 0 | 0 | Function unknown |
| OH11GL001757 | 81.50256 | 484.9418 | 2.572894 | 2.26E-12 | 9.72E-12 | Function unknown |
| OH11GL001758 | 6.625285 | 25.59228 | 1.949654 | 4.22E-14 | 2.27E-13 | Function unknown |
| OH11GL001771 | 88.64029 | 186.4676 | 1.07289 | 0 | 0 | Amino acid transport and metabolism |
| OH11GL001772 | 25.0001 | 71.56142 | 1.517248 | 4.25E-11 | 1.52E-10 | Function unknown |
| OH11GL001829 | 351.6523 | 157.0138 | -1.16326 | 1.55E-257 | 1.85E-256 | Energy production and conversion |
| OH11GL001839 | 81.30467 | 227.9582 | 1.487359 | 6.69E-13 | 3.09E-12 | Function unknown |
| OH11GL001887 | 54.38539 | 278.6559 | 2.357194 | 1.16E-12 | 5.19E-12 | Function unknown |
| OH11GL001926 | 0.846157 | 2.617513 | 1.629199 | 0.000231 | 0.000508 | Function unknown |
| OH11GL001932 | 27.37441 | 83.8956 | 1.615767 | 9.77E-15 | 5.36E-14 | Cell motility |
| OH11GL001933 | 50.76291 | 144.185 | 1.506075 | 0 | 0 | Posttranslational modification,protein turnover,chaperones |
| OH11GL001934 | 23.93058 | 58.2106 | 1.282427 | 0 | 0 | Function unknown |
| OH11GL001944 | 126.7396 | 58.72566 | -1.1098 | 1.04E-32 | 7.57E-32 | Function unknown |
| OH11GL001945 | 38.09539 | 7.440452 | -2.35615 | 9.14E-12 | 3.53E-11 | Function unknown |
| OH11GL001946 | 38.3066 | 11.4807 | -1.73838 | 1.12E-11 | 4.28E-11 | Replication, recombination and repair |
| OH11GL001951 | 62.94668 | 25.42154 | -1.30808 | 2.34E-11 | 8.59E-11 | Function unknown |
| OH11GL001967 | 1672.161 | 6894.244 | 2.043679 | 0 | 0 | Function unknown |
| OH11GL001975 | 2703.924 | 1132.847 | -1.2551 | 0 | 0 | Function unknown |
| OH11GL002061 | 6.016973 | 2.883701 | -1.06112 | 6.11E-05 | 0.000143 | Function unknown |
| OH11GL002092 | 18.95981 | 42.4705 | 1.163517 | 9.10E-15 | 5.02E-14 | Transcription |
| OH11GL002113 | 11.52487 | 5.540762 | -1.05659 | 0.000132 | 0.000299 | Function unknown |
| OH11GL002133 | 6.250025 | 14.82923 | 1.24651 | 0 | 0 | Function unknown |
| OH11GL002138 | 28.94378 | 8.851693 | -1.70923 | 2.48E-44 | 2.01E-43 | Function unknown |
| OH11GL002145 | 121.1543 | 388.0864 | 1.679532 | 4.78E-13 | 2.26E-12 | Function unknown |
| OH11GL002194 | 26.3659 | 58.59872 | 1.152196 | 4.82E-13 | 2.28E-12 | Secondary metabolites biosynthesis, transport and catabolism |
| OH11GL002202 | 10.49873 | 22.8291 | 1.12066 | 8.28E-14 | 4.35E-13 | Function unknown |
| OH11GL002209 | 6.395374 | 0.403745 | -3.98551 | 0.000363 | 0.000779 | Function unknown |
| OH11GL002216 | 285.246 | 634.3941 | 1.153173 | 0 | 0 | Inorganic ion transport and metabolism |
| OH11GL002234 | 23.93626 | 11.94342 | -1.00298 | 8.95E-14 | 4.67E-13 | Function unknown |
| OH11GL002281 | 60.31274 | 26.90964 | -1.16434 | 1.03E-12 | 4.65E-12 | Function unknown |
| OH11GL002294 | 27.39594 | 138.8884 | 2.341892 | 0 | 0 | Function unknown |
| OH11GL002295 | 15.1126 | 71.62133 | 2.244637 | 3.26E-14 | 1.76E-13 | Function unknown |
| OH11GL002296 | 5.21791 | 31.89497 | 2.611785 | 0 | 0 | Function unknown |
| OH11GL002297 | 24.55762 | 109.829 | 2.161016 | 0 | 0 | Function unknown |
| OH11GL002298 | 22.92462 | 111.7004 | 2.284664 | 0 | 0 | Function unknown |
| OH11GL002299 | 32.38525 | 191.5794 | 2.564534 | 3.68E-13 | 1.78E-12 | Function unknown |
| OH11GL002300 | 53.76859 | 272.2239 | 2.339958 | 7.99E-13 | 3.66E-12 | Function unknown |
| OH11GL002301 | 20.02796 | 124.2703 | 2.633394 | 0 | 0 | Function unknown |
| OH11GL002302 | 16.84499 | 95.29084 | 2.500018 | 4.78E-13 | 2.26E-12 | Function unknown |
| OH11GL002303 | 40.10432 | 117.5488 | 1.55143 | 2.44E-13 | 1.21E-12 | Function unknown |
| OH11GL002304 | 15.64272 | 67.31087 | 2.105348 | 0 | 0 | Amino acid transport and metabolism |
| OH11GL002317 | 15.60351 | 38.49365 | 1.30275 | 1.14E-12 | 5.11E-12 | Cell motility |
| OH11GL002318 | 6.854866 | 21.93467 | 1.678012 | 0 | 0 | Function unknown |
| OH11GL002325 | 3.520422 | 7.83019 | 1.153299 | 8.71E-10 | 2.86E-09 | Function unknown |
| OH11GL002370 | 343.8763 | 892.2714 | 1.375593 | 0 | 0 | Function unknown |
| OH11GL002371 | 158.9578 | 364.3965 | 1.196865 | 2.19E-12 | 9.45E-12 | Function unknown |
| OH11GL002411 | 5.418272 | 20.92568 | 1.94937 | 0 | 0 | Function unknown |
| OH11GL002419 | 4.649909 | 1.937448 | -1.26304 | 4.48E-07 | 1.24E-06 | Energy production and conversion |
| OH11GL002428 | 47.58273 | 109.4483 | 1.20174 | 5.39E-13 | 2.54E-12 | Function unknown |
| OH11GL002429 | 12.77327 | 79.00229 | 2.628766 | 6.00E-14 | 3.17E-13 | Function unknown |
| OH11GL002430 | 4.182173 | 15.03764 | 1.846254 | 0 | 0 | Function unknown |
| OH11GL002431 | 4.440172 | 33.04633 | 2.895803 | 0 | 0 | Function unknown |
| OH11GL002432 | 3.617035 | 24.87187 | 2.781635 | 0 | 0 | Nucleotide transport and metabolism |
| OH11GL002433 | 2.678582 | 8.680528 | 1.696313 | 6.19E-08 | 1.82E-07 | Function unknown |
| OH11GL002434 | 1.700259 | 12.33203 | 2.858585 | 3.59E-13 | 1.74E-12 | Function unknown |
| OH11GL002435 | 4.249565 | 20.24933 | 2.252487 | 0 | 0 | Function unknown |
| OH11GL002436 | 3.125012 | 11.30487 | 1.855011 | 5.62E-14 | 2.98E-13 | Function unknown |
| OH11GL002437 | 15.79541 | 118.9674 | 2.912989 | 0 | 0 | Secondary metabolites biosynthesis, transport and catabolism |
| OH11GL002438 | 6.57543 | 53.43901 | 3.022736 | 5.10E-13 | 2.41E-12 | Secondary metabolites biosynthesis, transport and catabolism |
| OH11GL002440 | 2.55306 | 13.25104 | 2.375806 | 2.44E-13 | 1.21E-12 | Secondary metabolites biosynthesis, transport and catabolism |
| OH11GL002441 | 8.059242 | 16.90419 | 1.068664 | 1.13E-13 | 5.85E-13 | Function unknown |
| OH11GL002442 | 13.97344 | 73.93301 | 2.403532 | 1.13E-13 | 5.85E-13 | Function unknown |
| OH11GL002443 | 8.500033 | 39.04308 | 2.199527 | 2.11E-14 | 1.15E-13 | Carbohydrate transport and metabolism |
| OH11GL002444 | 0.568184 | 7.299535 | 3.683374 | 7.83E-05 | 0.000182 | Function unknown |
| OH11GL002446 | 66.02848 | 27.16165 | -1.28152 | 1.30E-45 | 1.07E-44 | Function unknown |
| OH11GL002461 | 25.55566 | 6.36572 | -2.00525 | 1.62E-13 | 8.25E-13 | Function unknown |
| OH11GL002497 | 6.172864 | 20.36173 | 1.721848 | 7.31E-08 | 2.14E-07 | Function unknown |
| OH11GL002498 | 21.52786 | 78.2855 | 1.86254 | 1.18E-14 | 6.44E-14 | Function unknown |
| OH11GL002514 | 149.4668 | 29.84124 | -2.32445 | 7.20E-265 | 8.66E-264 | Function unknown |
| OH11GL002515 | 1306.527 | 110.4566 | -3.56419 | 0 | 0 | Function unknown |
| OH11GL002516 | 244.3131 | 40.60099 | -2.58914 | 0 | 0 | Function unknown |
| OH11GL002517 | 986.9041 | 58.34781 | -4.08016 | 0 | 0 | Energy production and conversion |
| OH11GL002518 | 484.4596 | 29.39438 | -4.04276 | 0 | 0 | Energy production and conversion |
| OH11GL002519 | 117.8576 | 7.440452 | -3.98551 | 1.12E-55 | 9.68E-55 | Function unknown |
| OH11GL002520 | 81.18844 | 10.65728 | -2.92943 | 5.09E-69 | 4.62E-68 | Function unknown |
| OH11GL002521 | 374.7611 | 38.22771 | -3.29328 | 0 | 0 | Amino acid transport and metabolism |
| OH11GL002522 | 46.81837 | 17.99237 | -1.37969 | 2.62E-09 | 8.37E-09 | Function unknown |
| OH11GL002543 | 4661.959 | 1371.955 | -1.7647 | 0 | 0 | Cell wall/membrane/envelope biogenesis |
| OH11GL002553 | 36.64398 | 77.05455 | 1.072304 | 2.32E-12 | 9.97E-12 | Function unknown |
| OH11GL002571 | 3.838189 | 7.924133 | 1.045827 | 6.88E-05 | 0.00016 | Defense mechanisms |
| OH11GL002578 | 1.368619 | 0.570254 | -1.26304 | 0.000241 | 0.000528 | Function unknown |
| OH11GL002592 | 1145.625 | 318.2015 | -1.84812 | 0 | 0 | Function unknown |
| OH11GL002595 | 179.7801 | 597.9352 | 1.733756 | 3.34E-13 | 1.62E-12 | Inorganic ion transport and metabolism |
| OH11GL002596 | 4825.962 | 465.3636 | -3.37439 | 0 | 0 | Inorganic ion transport and metabolism |
| OH11GL002652 | 41.03322 | 83.39046 | 1.02309 | 5.47E-12 | 2.18E-11 | Replication, recombination and repair |
| OH11GL002698 | 72.59203 | 154.2585 | 1.087467 | 3.40E-12 | 1.42E-11 | Amino acid transport and metabolism |
| OH11GL002701 | 541.6198 | 1221.401 | 1.173185 | 2.15E-11 | 7.93E-11 | Function unknown |
| OH11GL002709 | 12.2642 | 2.948104 | -2.05659 | 7.23E-05 | 0.000168 | Function unknown |
| OH11GL002710 | 21.15393 | 10.11138 | -1.06495 | 2.83E-05 | 6.86E-05 | Function unknown |
| OH11GL002712 | 31.25012 | 13.02079 | -1.26304 | 4.65E-06 | 1.21E-05 | Function unknown |
| OH11GL002784 | 65.8276 | 27.39466 | -1.2648 | 9.99E-74 | 9.25E-73 | Function unknown |
| OH11GL002785 | 22.39195 | 9.630305 | -1.21733 | 1.96E-23 | 1.28E-22 | Function unknown |
| OH11GL002790 | 71.99102 | 156.8121 | 1.123148 | 0 | 0 | Function unknown |
| OH11GL002871 | 344.4592 | 866.7141 | 1.331223 | 0 | 0 | Function unknown |
| OH11GL002873 | 48.38254 | 119.587 | 1.305502 | 4.42E-13 | 2.11E-12 | Function unknown |
| OH11GL002881 | 955.8436 | 1985.768 | 1.05485 | 0 | 0 | Carbohydrate transport and metabolism |
| OH11GL002915 | 235.8218 | 81.88199 | -1.52608 | 3.70E-57 | 3.22E-56 | Function unknown |
| OH11GL002929 | 9.005412 | 24.40815 | 1.438499 | 0 | 0 | Function unknown |
| OH11GL002962 | 31.0346 | 62.26034 | 1.004436 | 1.03E-07 | 2.99E-07 | Function unknown |
| OH11GL002968 | 57.86075 | 118.0388 | 1.028604 | 2.84E-12 | 1.20E-11 | Function unknown |
| OH11GL002985 | 6.072375 | 14.26567 | 1.232215 | 0 | 0 | Function unknown |
| OH11GL002999 | 106.2504 | 319.8441 | 1.5899 | 5.08E-14 | 2.71E-13 | Function unknown |
| OH11GL003001 | 12.89479 | 38.19432 | 1.56657 | 0 | 0 | Function unknown |
| OH11GL003003 | 17.10533 | 61.82972 | 1.853855 | 6.04E-14 | 3.20E-13 | Function unknown |
| OH11GL003027 | 31.44666 | 11.13728 | -1.49751 | 1.01E-18 | 6.14E-18 | Function unknown |
| OH11GL003028 | 362.999 | 137.946 | -1.39586 | 0 | 0 | Lipid transport and metabolism |
| OH11GL003037 | 51.8914 | 143.62 | 1.468689 | 0 | 0 | Function unknown |
| OH11GL003060 | 11.97679 | 4.683447 | -1.3546 | 1.02E-16 | 5.96E-16 | Energy production and conversion |
| OH11GL003061 | 73.87227 | 35.25041 | -1.06739 | 8.81E-142 | 9.56E-141 | Energy production and conversion |
| OH11GL003062 | 29.38156 | 8.948997 | -1.71511 | 1.88E-25 | 1.28E-24 | Energy production and conversion |
| OH11GL003131 | 2.855257 | 1.406407 | -1.02161 | 1.91E-05 | 4.71E-05 | Function unknown |
| OH11GL003133 | 2.23098 | 0.546805 | -2.02858 | 6.65E-06 | 1.71E-05 | Inorganic ion transport and metabolism |
| OH11GL003169 | 8974.222 | 486.8153 | -4.20434 | 0 | 0 | Replication, recombination and repair |
| OH11GL003179 | 28.08653 | 11.78837 | -1.25251 | 2.18E-13 | 1.09E-12 | Function unknown |
| OH11GL003223 | 286.3671 | 1179.03 | 2.041663 | 0 | 0 | Function unknown |
| OH11GL003224 | 29.34953 | 92.08466 | 1.649623 | 0 | 0 | Function unknown |
| OH11GL003239 | 68.09419 | 149.0557 | 1.130248 | 3.13E-13 | 1.53E-12 | Function unknown |
| OH11GL003240 | 17.14292 | 34.32529 | 1.001658 | 6.75E-12 | 2.65E-11 | Function unknown |
| OH11GL003292 | 13.43675 | 44.72603 | 1.734931 | 4.45E-13 | 2.12E-12 | General function prediction only |
| OH11GL003311 | 22.6324 | 10.73581 | -1.07596 | 4.31E-18 | 2.59E-17 | Function unknown |
| OH11GL003312 | 22.4853 | 8.218251 | -1.45208 | 5.36E-14 | 2.85E-13 | Transcription |
| OH11GL003329 | 37.77793 | 90.77352 | 1.264728 | 6.00E-13 | 2.80E-12 | Function unknown |
| OH11GL003330 | 85.03555 | 177.6452 | 1.06286 | 0 | 0 | Function unknown |
| OH11GL003337 | 28.79846 | 13.18865 | -1.12669 | 2.01E-24 | 1.34E-23 | Inorganic ion transport and metabolism |
| OH11GL003361 | 19.96311 | 127.5283 | 2.67541 | 5.40E-13 | 2.54E-12 | Function unknown |
| OH11GL003365 | 24.20222 | 248.5955 | 3.360589 | 0 | 0 | Function unknown |
| OH11GL003366 | 70.86015 | 205.8999 | 1.538897 | 0 | 0 | Replication, recombination and repair |
| OH11GL003369 | 11.59425 | 24.15451 | 1.058883 | 9.62E-08 | 2.80E-07 | Function unknown |
| OH11GL003372 | 51.55461 | 125.5753 | 1.284379 | 5.48E-13 | 2.57E-12 | Cell wall/membrane/envelope biogenesis |
| OH11GL003390 | 464.9653 | 32.06093 | -3.85824 | 0 | 0 | Function unknown |
| OH11GL003391 | 970.7275 | 307.7019 | -1.65753 | 2.05E-293 | 2.51E-292 | Function unknown |
| OH11GL003394 | 32.46766 | 15.55731 | -1.06141 | 1.74E-11 | 6.52E-11 | Function unknown |
| OH11GL003405 | 173.0776 | 3169.482 | 4.194756 | 0 | 0 | Function unknown |
| OH11GL003406 | 304.9452 | 2229.017 | 2.869786 | 1.30E-11 | 4.92E-11 | General function prediction only |
| OH11GL003437 | 6497.036 | 2673.602 | -1.281 | 0 | 0 | Function unknown |
| OH11GL003464 | 13.70198 | 30.63225 | 1.160667 | 8.88E-14 | 4.65E-13 | Function unknown |
| OH11GL003496 | 45.97205 | 100.8362 | 1.133185 | 1.34E-12 | 5.95E-12 | Function unknown |
| OH11GL003529 | 16.69587 | 37.75726 | 1.177263 | 0 | 0 | General function prediction only |
| OH11GL003533 | 2.142866 | 6.24998 | 1.54431 | 5.39E-05 | 0.000127 | Carbohydrate transport and metabolism |
| OH11GL003553 | 2.853272 | 0.660475 | -2.11104 | 0.000293 | 0.000637 | Lipid transport and metabolism |
| OH11GL003579 | 1.555562 | 4.320974 | 1.473921 | 0.00024 | 0.000527 | Function unknown |
| OH11GL003599 | 42.10543 | 15.99045 | -1.3968 | 2.18E-06 | 5.80E-06 | Function unknown |
| OH11GL003646 | 83.65417 | 177.2831 | 1.083545 | 1.92E-13 | 9.69E-13 | Function unknown |
| OH11GL003653 | 267.6293 | 104.3147 | -1.35929 | 2.37E-185 | 2.69E-184 | Signal transduction mechanisms |
| OH11GL003667 | 10.00004 | 24.15451 | 1.272287 | 1.88E-08 | 5.72E-08 | Function unknown |
| OH11GL003702 | 112.6968 | 51.62869 | -1.1262 | 1.36E-48 | 1.13E-47 | Function unknown |
| OH11GL003703 | 174.2984 | 54.91165 | -1.66638 | 1.57E-125 | 1.67E-124 | Function unknown |
| OH11GL003722 | 6.594514 | 13.80682 | 1.066043 | 3.75E-08 | 1.12E-07 | Function unknown |
| OH11GL003733 | 23.67031 | 717.6211 | 4.922072 | 1.82E-13 | 9.23E-13 | Function unknown |
| OH11GL003786 | 4.408078 | 17.75463 | 2.009974 | 0 | 0 | Lipid transport and metabolism |
| OH11GL003787 | 7.887731 | 25.12866 | 1.671651 | 0 | 0 | Lipid transport and metabolism |
| OH11GL003834 | 4.761923 | 18.35312 | 1.946409 | 3.09E-10 | 1.04E-09 | Function unknown |
| OH11GL003835 | 21.80143 | 66.57994 | 1.610665 | 0 | 0 | Amino acid transport and metabolism |
| OH11GL003836 | 15.59026 | 48.10056 | 1.625409 | 0 | 0 | Amino acid transport and metabolism |
| OH11GL003837 | 16.79593 | 53.65329 | 1.675555 | 2.38E-13 | 1.19E-12 | Amino acid transport and metabolism |
| OH11GL003838 | 46.03193 | 119.3687 | 1.374718 | 3.55E-13 | 1.72E-12 | Amino acid transport and metabolism |
| OH11GL003839 | 65.10093 | 195.9818 | 1.58997 | 3.06E-13 | 1.50E-12 | Amino acid transport and metabolism |
| OH11GL003840 | 51.4883 | 152.047 | 1.562201 | 0 | 0 | Amino acid transport and metabolism |
| OH11GL003841 | 32.51001 | 73.90457 | 1.18478 | 4.42E-13 | 2.11E-12 | Amino acid transport and metabolism |
| OH11GL003842 | 44.15155 | 116.0324 | 1.393991 | 7.62E-13 | 3.50E-12 | Amino acid transport and metabolism |
| OH11GL003887 | 36.00338 | 81.9734 | 1.187024 | 2.34E-12 | 1.00E-11 | Function unknown |
| OH11GL003895 | 64.22345 | 30.8852 | -1.05618 | 3.27E-59 | 2.87E-58 | Translation, ribosomal structure and biogenesis |
| OH11GL003914 | 2.678582 | 5.855911 | 1.128424 | 0.000208 | 0.000459 | Function unknown |
| OH11GL003960 | 88.9244 | 249.2081 | 1.486699 | 0 | 0 | Function unknown |
| OH11GL003962 | 100.7046 | 289.5139 | 1.523503 | 0 | 0 | Function unknown |
| OH11GL004007 | 26.02951 | 12.45911 | -1.06295 | 5.26E-28 | 3.67E-27 | Inorganic ion transport and metabolism |
| OH11GL004008 | 79.51573 | 37.78133 | -1.07357 | 4.61E-38 | 3.53E-37 | Inorganic ion transport and metabolism |
| OH11GL004023 | 5.833356 | 20.83327 | 1.836491 | 1.07E-06 | 2.91E-06 | Function unknown |
| OH11GL004076 | 4.489282 | 24.81603 | 2.466716 | 0 | 0 | Secondary metabolites biosynthesis, transport and catabolism |
| OH11GL004077 | 4.645779 | 27.96723 | 2.589745 | 0 | 0 | Secondary metabolites biosynthesis, transport and catabolism |
| OH11GL004079 | 7.785767 | 31.8295 | 2.031453 | 2.08E-12 | 9.00E-12 | Secondary metabolites biosynthesis, transport and catabolism |
| OH11GL004120 | 291.7753 | 99.23803 | -1.55589 | 0 | 0 | Lipid transport and metabolism |
| OH11GL004121 | 237.0782 | 66.67652 | -1.83011 | 1.85E-225 | 2.19E-224 | Transcription |
| OH11GL004185 | 77.98326 | 762.3081 | 3.289138 | 9.93E-14 | 5.15E-13 | Function unknown |
| OH11GL004187 | 15.00006 | 43.48531 | 1.53556 | 0 | 0 | Function unknown |
| OH11GL004192 | 165.0006 | 63.61091 | -1.37513 | 1.97E-63 | 1.74E-62 | Function unknown |
| OH11GL004245 | 36.5386 | 15.9143 | -1.1991 | 2.16E-15 | 1.21E-14 | Function unknown |
| OH11GL004275 | 35.15453 | 12.3713 | -1.50671 | 1.15E-52 | 9.71E-52 | Function unknown |
| OH11GL004276 | 201.2934 | 52.91509 | -1.92755 | 0 | 0 | Inorganic ion transport and metabolism |
| OH11GL004296 | 125.9074 | 312.8276 | 1.313004 | 9.49E-12 | 3.66E-11 | Amino acid transport and metabolism |
| OH11GL004337 | 12.04824 | 30.43414 | 1.336868 | 0 | 0 | Function unknown |
| OH11GL004346 | 43.75017 | 742.877 | 4.085763 | 0 | 0 | Function unknown |
| OH11GL004351 | 145.8984 | 71.70169 | -1.02488 | 1.88E-89 | 1.83E-88 | Secondary metabolites biosynthesis, transport and catabolism |
| OH11GL004387 | 3.360781 | 7.311171 | 1.121306 | 1.42E-12 | 6.29E-12 | Function unknown |
| OH11GL004414 | 39.22117 | 181.7879 | 2.212551 | 1.87E-12 | 8.10E-12 | Posttranslational modification,protein turnover,chaperones |
| OH11GL004442 | 20.63115 | 9.607574 | -1.10258 | 1.57E-10 | 5.40E-10 | Energy production and conversion |
| OH11GL004466 | 21.75009 | 44.61791 | 1.036602 | 1.25E-09 | 4.06E-09 | Function unknown |
| OH11GL004502 | 126.5492 | 271.7466 | 1.102564 | 0 | 0 | Function unknown |
| OH11GL004503 | 126.4921 | 290.2901 | 1.198448 | 0 | 0 | Energy production and conversion |
| OH11GL004508 | 18.59144 | 37.45405 | 1.010483 | 5.10E-13 | 2.41E-12 | Function unknown |
| OH11GL004510 | 22.3388 | 54.71533 | 1.292393 | 0 | 0 | Energy production and conversion |
| OH11GL004542 | 3.095987 | 0.752492 | -2.04065 | 9.15E-07 | 2.51E-06 | Function unknown |
| OH11GL004565 | 26.52593 | 76.37234 | 1.525647 | 9.10E-15 | 5.01E-14 | Function unknown |
| OH11GL004574 | 3.463869 | 34.66982 | 3.323224 | 0 | 0 | Lipid transport and metabolism |
| OH11GL004575 | 5.357164 | 26.18331 | 2.289106 | 0 | 0 | Function unknown |
| OH11GL004583 | 14.49586 | 30.92894 | 1.093317 | 8.72E-09 | 2.72E-08 | Function unknown |
| OH11GL004586 | 5.501952 | 15.93106 | 1.533827 | 3.80E-13 | 1.83E-12 | Function unknown |
| OH11GL004621 | 1.701577 | 3.726719 | 1.131033 | 0.000264 | 0.000577 | Function unknown |
| OH11GL004642 | 117.1953 | 56.28079 | -1.0582 | 1.03E-99 | 1.03E-98 | Carbohydrate transport and metabolism |
| OH11GL004650 | 71.61793 | 35.5391 | -1.01091 | 6.91E-24 | 4.55E-23 | Function unknown |
| OH11GL004651 | 117.0087 | 46.39102 | -1.3347 | 1.63E-42 | 1.31E-41 | Function unknown |
| OH11GL004681 | 2.019239 | 0.767893 | -1.39483 | 0.000128 | 0.00029 | Intracellular trafficking, secretion, and vesicular transport |
| OH11GL004683 | 204.652 | 98.71577 | -1.05182 | 5.60E-36 | 4.23E-35 | Function unknown |
| OH11GL004695 | 74.10743 | 304.0251 | 2.0365 | 0 | 0 | Function unknown |
| OH11GL004696 | 83.88191 | 258.1315 | 1.621674 | 0 | 0 | Function unknown |
| OH11GL004805 | 47.18562 | 18.16587 | -1.37712 | 4.52E-23 | 2.95E-22 | Function unknown |
| OH11GL004806 | 301.1518 | 109.0332 | -1.46572 | 1.20E-238 | 1.43E-237 | Transcription |
| OH11GL004809 | 3.189313 | 7.608857 | 1.254434 | 1.30E-10 | 4.50E-10 | Replication, recombination and repair |
| OH11GL004816 | 31.119 | 204.3263 | 2.715007 | 1.82E-13 | 9.23E-13 | Function unknown |
| OH11GL004819 | 104.7462 | 272.7157 | 1.3805 | 0 | 0 | Function unknown |
| OH11GL004820 | 115.5284 | 250.3155 | 1.1155 | 0 | 0 | Function unknown |
| OH11GL004829 | 13.03035 | 5.6818 | -1.19746 | 1.14E-06 | 3.09E-06 | Function unknown |
| OH11GL004830 | 13.04883 | 3.810963 | -1.77569 | 2.53E-13 | 1.25E-12 | Function unknown |
| OH11GL004869 | 659.9733 | 196.8774 | -1.74511 | 0 | 0 | Amino acid transport and metabolism |
| OH11GL004870 | 125.377 | 52.03087 | -1.26883 | 2.30E-111 | 2.36E-110 | Energy production and conversion |
| OH11GL004871 | 170.0141 | 70.23974 | -1.2753 | 4.60E-169 | 5.12E-168 | Amino acid transport and metabolism |
| OH11GL004891 | 25.98049 | 66.04009 | 1.345913 | 9.77E-11 | 3.43E-10 | Function unknown |
| OH11GL004896 | 19.34939 | 88.80978 | 2.19843 | 7.59E-14 | 4.00E-13 | Defense mechanisms |
| OH11GL004897 | 3.641471 | 45.71258 | 3.649998 | 0 | 0 | Cell wall/membrane/envelope biogenesis |
| OH11GL004898 | 20.78596 | 222.0554 | 3.417239 | 2.31E-13 | 1.16E-12 | Amino acid transport and metabolism |
| OH11GL004899 | 13.25884 | 129.4591 | 3.28747 | 2.42E-13 | 1.20E-12 | Function unknown |
| OH11GL004900 | 11.15061 | 125.8676 | 3.496713 | 8.93E-14 | 4.67E-13 | Defense mechanisms |
| OH11GL004901 | 24.76276 | 245.7468 | 3.310929 | 0 | 0 | Defense mechanisms |
| OH11GL004902 | 9.662066 | 121.5205 | 3.652724 | 0 | 0 | Secondary metabolites biosynthesis,transport and catabolism |
| OH11GL004903 | 37.76056 | 325.8815 | 3.109395 | 3.55E-15 | 1.98E-14 | Function unknown |
| OH11GL004904 | 39.51823 | 522.2958 | 3.724277 | 7.29E-13 | 3.35E-12 | Coenzyme transport and metabolism |
| OH11GL004905 | 14.27398 | 246.0342 | 4.107399 | 3.44E-13 | 1.66E-12 | Lipid transport and metabolism |
| OH11GL004906 | 16.96934 | 266.9626 | 3.975635 | 0 | 0 | Carbohydrate transport and metabolism |
| OH11GL004907 | 9.670917 | 154.6778 | 3.999469 | 0 | 0 | Secondary metabolites biosynthesis,transport and catabolism |
| OH11GL004908 | 1.465523 | 25.32319 | 4.110972 | 7.25E-10 | 2.39E-09 | Coenzyme transport and metabolism |
| OH11GL004925 | 51.53081 | 130.7394 | 1.343186 | 5.40E-14 | 2.87E-13 | Function unknown |
| OH11GL004926 | 3.7736 | 24.23996 | 2.683374 | 1.04E-06 | 2.84E-06 | Function unknown |
| OH11GL004927 | 39.07119 | 259.8466 | 2.733483 | 0 | 0 | Function unknown |
| OH11GL004939 | 63.37234 | 167.9581 | 1.406176 | 7.50E-13 | 3.44E-12 | Function unknown |
| OH11GL004947 | 5.60944 | 13.22518 | 1.237359 | 5.46E-13 | 2.56E-12 | Function unknown |
| OH11GL004966 | 4.27854 | 1.572981 | -1.44362 | 1.46E-05 | 3.63E-05 | Carbohydrate transport and metabolism |
| OH11GL004967 | 33.45084 | 8.436006 | -1.98741 | 5.17E-26 | 3.53E-25 | Function unknown |
| OH11GL004968 | 200.705 | 47.80403 | -2.06987 | 1.37E-155 | 1.51E-154 | Amino acid transport and metabolism |
| OH11GL004980 | 9.106927 | 19.09472 | 1.068137 | 0 | 0 | Function unknown |
| OH11GL005002 | 28.70979 | 58.7289 | 1.032528 | 0 | 0 | Function unknown |
| OH11GL005007 | 23.26932 | 51.7493 | 1.15311 | 2.96E-13 | 1.46E-12 | Function unknown |
| OH11GL005011 | 67.40026 | 147.3954 | 1.128865 | 1.27E-12 | 5.68E-12 | Function unknown |
| OH11GL005012 | 162.0006 | 574.1301 | 1.825378 | 0 | 0 | Function unknown |
| OH11GL005092 | 1.125004 | 5.208317 | 2.210886 | 1.05E-06 | 2.85E-06 | Inorganic ion transport and metabolism |
| OH11GL005093 | 425.6596 | 1910.173 | 2.165931 | 4.16E-12 | 1.70E-11 | Function unknown |
| OH11GL005094 | 14.04115 | 87.99439 | 2.64775 | 9.15E-14 | 4.77E-13 | Function unknown |
| OH11GL005109 | 644.3169 | 318.3634 | -1.0171 | 0 | 0 | Amino acid transport and metabolism |
| OH11GL005112 | 1042.384 | 520.0587 | -1.00314 | 0 | 0 | Lipid transport and metabolism |
| OH11GL005113 | 2849.841 | 1283.138 | -1.1512 | 0 | 0 | Secondary metabolites biosynthesis,transport and catabolism |
| OH11GL005114 | 2354.933 | 974.1645 | -1.27345 | 0 | 0 | Secondary metabolites biosynthesis,transport and catabolism |
| OH11GL005115 | 1416.543 | 632.3742 | -1.16352 | 0 | 0 | Secondary metabolites biosynthesis,transport and catabolism |
| OH11GL005116 | 2037.112 | 850.8445 | -1.25956 | 0 | 0 | Secondary metabolites biosynthesis,transport and catabolism |
| OH11GL005117 | 1896.953 | 649.412 | -1.54648 | 0 | 0 | Function unknown |
| OH11GL005118 | 171.3789 | 80.5525 | -1.08919 | 7.72E-177 | 8.68E-176 | Function unknown |
| OH11GL005119 | 636.9331 | 205.6588 | -1.63089 | 0 | 0 | Function unknown |
| OH11GL005120 | 378.5868 | 119.1049 | -1.66839 | 0 | 0 | Amino acid transport and metabolism |
| OH11GL005121 | 81.89182 | 25.9143 | -1.65997 | 1.47E-112 | 1.52E-111 | Function unknown |
| OH11GL005127 | 11.71176 | 28.54408 | 1.285234 | 0 | 0 | Function unknown |
| OH11GL005128 | 87.2593 | 214.7893 | 1.299541 | 4.24E-12 | 1.72E-11 | Function unknown |
| OH11GL005147 | 13.55938 | 236.7577 | 4.126049 | 8.28E-14 | 4.35E-13 | Function unknown |
| OH11GL005162 | 12.32399 | 47.68177 | 1.951968 | 3.22E-14 | 1.74E-13 | Function unknown |
| OH11GL005163 | 24.68364 | 102.4082 | 2.052705 | 0 | 0 | Function unknown |
| OH11GL005164 | 89.89006 | 216.1196 | 1.265596 | 3.34E-13 | 1.63E-12 | Posttranslational modification, protein turnover, chaperones |
| OH11GL005165 | 82.59056 | 175.6747 | 1.088858 | 3.73E-12 | 1.54E-11 | Posttranslational modification, protein turnover, chaperones |
| OH11GL005168 | 9.343127 | 27.77296 | 1.571704 | 9.93E-14 | 5.15E-13 | Function unknown |
| OH11GL005230 | 34.19368 | 69.55623 | 1.02445 | 0 | 0 | Function unknown |
| OH11GL005250 | 380.6406 | 83.71596 | -2.18485 | 0 | 0 | Inorganic ion transport and metabolism |

^a^ The expression of genes with shadows was controlled by lesR and also influenced by Clp.
